# Clinicopathological study of otomycosis in a tertiary hospital in South-west Nigeria

**DOI:** 10.4314/ahs.v24i1.9

**Published:** 2024-03

**Authors:** Olusola A Sogebi, Emmanuel A Oyewole, Olubunmi A Osinupebi

**Affiliations:** 1 Department of Surgery, Obafemi Awolowo College of Health Sciences, Olabisi Onabanjo University, Sagamu, Nigeria; 2 Department of Medical Microbiology and Parasitology, Olabisi Onabanjo University Teaching Hospital, Sagamu, Nigeria

**Keywords:** Otomycosis, mycology, presentations, treatment, complications, associations

## Abstract

**Background:**

Otomycosis is common in environments with hot, humid weather, and it may be challenging to manage.

**Objectives:**

To profile common clinical presentations, the pathogenic fungi, the treatment modalities with responses, and explore clinical factors associated with having positive fungal culture in Otomycosis.

**Methods:**

Retrospective review of patients with Otomycosis. Demographic and clinical parameters, otoscopic findings and mycological study results were recorded. The treatment modalities used and treatment response were summarized. Comparative statistical analyses of associated factors to positive fungal culture were performed with Chi square test, and Student's t-test, using SPSS version 22.0

**Results:**

Total of 71 patients with M: F=1:1.8, mean age 38.5±19.8 years. Average duration of symptoms was 5.4 ±4.6 weeks; common presenting complaint was itchy ear (33.8%). Majority of patients (85.9%) had unilateral ear involvement, 50.0% applied ototopic medications before presentation, 8.5% had multiple co-morbidities. 20 patients had positive fungal culture results; common fungal isolate was Aspergillus niger 9 (45.0%).

Clinical factors associated with positive culture of fungus were age, non-previous use of ototopic drugs, and presence of co-morbidity. The most common treatment was local ear debridement and use of topical antifungal creams. Majority (91.5%) of the patients responded with resolution of fungal infection. Complications rate was 8.4%.

**Conclusions:**

Otomycosis commonly present with itchy ears, the pathogenic fungi commonly being Aspergillus species. The factors associated with positive fungal culture were age, non-usage of ototopic agents and presence of co-morbidity. Treatment modality used was local debridement and topical antifungal agents, which produced favourable response in most patients.

## Introduction

Otomycosis, also called fungal otitis externa is a fungal infection of the external ear whose prevalence ranges between 25.0 and 39.6%.^1^,^2^ It is usually a superficial infection,^3^ and is responsible for 7-15% of otitis externa.^4^ Save for immune suppressive states such as diabetes mellitus, a normal ear canal is naturally protected against proliferation of organisms including fungi by presence of an intact canal skin, an acidic pH and presence of lysosomes in cerumen. Sometimes the infection can become invasive and spread into the middle ear cavity in presence of a non-intact tympanic membrane.

Patients with other chronic otorhinolaryngological lesions like chronic suppurative otitis media, and necrotizing otitis externa, (which may affect local immunity) are also predisposed to developing Otomycosis.^5^ In fact, analysis of the prevalence of mycoses based on the literature data and original observations gives evidence of the increase in the relative frequency of mycotic lesions in the overall ear, nose and throat (ENT) region.^1^ Notwithstanding the worldwide distribution, Otomycosis is common in environments with hot and humid weather conditions, typified in the tropical and subtropical regions of sub-Saharan Africa.^6^

Studies done in Iran 7 and south-eastern Serbia, 8 have reported Otomycosis to be more common among females and young adults, with its prevalence increasing with age.^8^ The fungal agent can either be a primary pathogen occurring de-novo, or a secondary infection.^6^,^7^ The clinical features are usually predictive of Otomycosis which can present as an acute, subacute or chronic superficial mycotic infection and can thus be reasonably diagnosed clinically.^7^ In low-resource settings, where diagnostic capacity is limited, clinical diagnosis is more often used than laboratory diagnosis of Otomycosis, Clinical diagnosis is based entirely on non-specific clinical signs and symptoms,^4^ which include persistently itchy ears, feeling of ear blockage, otorrhoea, and otalgia, and may demonstrate subtle variations in presentation according to the locality.

The diagnostic criteria for Otomycosis are based on any two or more of the following findings, namely the presence of symptoms and otoscopy findings, the demonstration of fungal elements in 10% Potassium hydroxide (KOH) preparation or culture of the clinical material (debris, discharge) on Saboraud Dextrose Agar (SDA).^9^ Different species of fungi are cultured in different geographical areas in the world. The main treatment is with antifungal agents, while ancillary treatment like debridement, ear canal acidification, treatment for pruritus and pain are added when necessary.

Although the disease is hardly life threatening, it can present a challenging and frustrating situation for the Otologist and patients, due to long term treatment that may be required and high rate of recurrence.^7^ In order to avoid these frustrations, it is expedient to confirm and characterize the fungal agents involved in order to guide appropriate drug selection which leads to reduction of treatment duration, and incidence of recurrence. Management of Otomycosis should be pain-staking, and may require a long follow-up period. 10

This study aimed to profile the common clinical presentations, and characterize the pathogenic strain of fungi seen in patients with Otomycosis in the study centre. We also explored the clinical factors that were associated with positive fungal culture, documented the treatment modalities used and the responses to treatment.

## Patients and methods

This retrospective cross-sectional, analytical study, utilized historical data (records) of patients with clinical diagnosis of Otomycosis who were managed in the Ear, Nose and Throat department of a tertiary hospital in South-western region of Nigeria. The hospital is located in an urban area and serves as a referral center for primary and secondary health facilities within the locality, surrounding towns and adjoining states. The patients were managed within the period of five years, ranging from 1^st^ January 2016 to 31^st^ December 2020. Ethics approval to conduct the study was granted by the institution's Health Research Ethics Committee, approval number OOUTH/HREC/384/2020AP.

All patients that were diagnosed with Otomycosis during the period of study were eligible, and their clinical records were included in the study. Records of patients with incomplete vital clinical information on the symptoms, signs, treatment modalities and possible outcome of treatment were excluded. Since the patients' information was used retrospectively, a waiver of patient's consent was obtained from the ethics committee.

Case note records of the patients seen during the study period were used to acquire information on basic demographic and clinical parameters including age, sex, the major presenting complaints, duration of symptoms, other symptoms, and presence of chronic medical condition(s). Chronic medical conditions that the patients had apart from Otomycosis were regarded as co-morbidities. The otoscopic findings in the pinna, external auditory canals, the state of the tympanic membrane, and hearing status were recorded. The major and any other diagnoses were noted. The available laboratory results of the ear swab for mycological studies were recorded.

Based on the laboratory standard operating procedure used at the current study site, ear specimens were aseptically-collected and immediately transported to the laboratory where a Potassium hydroxide (KOH) wet preparation was prepared and microscopically examined for fungal hyphae and yeast cells. Inoculation on to Sabouraud Dextrose Agar (SDA) containing 0.5mg/ml of Chloramphenicol was done in duplicate and incubated in air at 37 0C, and 25-27 0C respectively for 2-3weeks. Plates were checked on alternate days for visible colonies and those with colonies were characterized for colonial morphology, and microscopy performed. The germ tube biochemical test for Candida species was not performed. Some of the patients had characterization of the debris and the culture results noted.

The treatment for Otomycosis was summarized and the outcome was noted. Complications observed were noted. The number of patients that had adequate information required for the study at specific period constituted the sample size. A patient qualified to have had Otomycosis by clinical evaluation, and positive fungal culture by laboratory fungal culture. The data generated was input into the computer and results were presented in tabular format. The data was described and summarized with means and standard deviations for continuous variables, and proportions for categorical variables. In exploring the clinical factors only patients that had fungal culture results (Negative or Positive) were considered. The mean ages of the patients with and without culture positive swabs were calculated and compared using independent samples t-test. For all the other parameters, proportions of patients with and without positive culture results were calculated and compared using Chi-square tests. P-value of <0.05 was considered statistically significant. Data analyses were performed using SPSS version 22.0.

## Results

There were seventy-one eligible patients who were diagnosed clinically with Otomycosis within the study period, comprising 25 Males and 46 Females; M: F=1:1.8. The age ranged from 2 to 87 years with a mean age of 38.5±19.8 years. The patients' duration of symptoms ranged 1-24 weeks, with an average of 5.4 ±4.6 weeks. The patients presenting complaints were varied and multiple but the three most common ones were itchy ear (33.8%), feeling of ear blockage or hearing loss (25.4%), and earache (21.1%). The left ear was affected in 36 (50.7%) of the patients while both ears were involved in 10 (14.1%). Thirty three out of 48 patients (68.7%) admitted to habitual cleaning of the ears, while 22/44 patients (50.0%) had previously use antibiotic/steroid/antifungal eardrops (ototopic drugs) before presentation. Thirty-nine (54.9%) patients had no associated co-morbidities, while 6 (8.5%) had multiple co-morbidities. The associated co-morbid conditions seen in the patients included hypertension 6 (8.5%), diabetes mellitus 8 (11.3%), sickle cell disease 1 (1.4%), allergies 8 (11.3%), 3 (4.2%) patients were pregnant. A detail of the clinical characteristics of the patients is shown in Table 1.

**Table 1:** Clinical characteristics of the patients

Characteristic	Frequency (n)	Percentage (%)		
Age group (Years)				
1-15	8	11.3		
16-40	32	45.1		
41-60	19	26.8		
≥61	12	16.9		
Range = 2-87;	Mean ±Standard deviation= 38.5 ±19.8;			Median=39
Sex: Male			25	35.2
Female			46	64.8
Major/Presenting complaints				
Earache			15	21.1
Itchy ear			24	33.8
Ear blockage/hearing loss			18	25.4
Affected (side of) ear				
Right			25	35.2
Left			36	50.7
Both			10	14.1
*Habitual cleaning of the ear (48/71 responses)				
No			15	31.3
Yes			33	68.7
*Previous use of Ototopical drugs (44/71 responses)				
No			22	50.0
Yes			22	50.0
Co-morbidities				
Nil			39	54.9
Single			26	36.6
Multiple			6	8.5

* Missing data

Table 2 represents the mycopathological profile of the patients' ear swabs sent after laboratory analyses. The results of fungal studies were seen in 35 patients, with 20 being culture positive. The two common fungal isolates were Aspergillus niger 9 (45.0%), and Candida species in 5 (25.0%) specimens.

**Table 2:** Mycopathological profile of the patients' ear swabs

Parameter	Frequency (n)	Percentage (%)
Culture		
Not available	36	50.7
Negative	15	21.1
Positive	20	28.1
Isolated fungi (in 20 culture Positive swabs)		
Aspergillus niger	9	45.0
Aspergillus flavus	3	15.0
Aspergillus fumigatus	2	10.0
Candida species	5	25.0
Penicillium	1	5.0
Multiple organisms	2	10.0

In Table 3, the clinical factors that could be associated with positive fungal culture were explored. There were significant association with the mean age, age about the median of 39 years, non-previous use of ototopic drugs, and presence of co-morbidity. Multiple compared with single co-morbidity did not have any association with positive culture result, neither did sex, laterality i.e., right versus left, unilateral versus bilateral ear involvement, nor habitual cleaning of the ear.

**Table 3:** Clinical factors associated with positive culture for fungus

Factor	Positive Culture (%)		Statistic	p-Value
	No	Yes		
*Mean Age	25.9 ±17.3	48.4 ±20.5	3.440	0.002
Age >39 years	20.0	75.0	10.388	0.006
Sex: Female	53.3	70.0	1.157	0.561
Affected both ears	20.0	10.0	6.191	0.182
Habitual ear cleaning	46.7	45.0	1.054	0.900
No use of ototopic drugs	33.3	65.0	27.443	<0.001
Co-morbidity	33.3	70.0	20.510	0.016
Multiple co-morbidities	6.7	20.0	9.239	0.056

* The statistical test used was independent samples t-test

### Treatment modalities

The treatment modality mostly used was local ear debridement and use of topical antifungal creams in 46 (67.6%) patients, while 17 (23.9%) and 6 (8.5%) patients respectively had local debridement and topical antifungals alone. Majority (91.5%) of the patients responded with resolution of fungal infection. Four patients (5.6%) had recurrence of symptoms while the lesion did not resolve during treatment in 2 (2.8%) patients.

## Discussion

Otomycosis may be relatively easy to diagnose clinically from the pattern of presentation, but it may be difficult to treat. The age group of 20-40 years had been noted to have the highest predisposition,^7^,^11^ and the mean age of 38 years found in this study was similar to the average of 37 years recorded in a study in Khouzestan Province, south-west Iran.^11^ The pediatric age group had also been noted to have the lowest prevalence of Otomycosis,^7^ similar to the trend observed in this study.

The common clinical presentations of the patients in this study were similar to what was reported in other studies.^12^ However unlike what was found in Ibadan,^2^ which is located about 60km from the present study centre, otorrhoea was not a prominent presenting symptom in our patients. The mean duration of symptoms before presentation of 5 weeks was rather late. This late presentation might be connected with the patients' practice of applying un-prescribed off- the- shelf ototopic medications, and present to hospital when symptoms did not abate. While close to a third (31.0%) of our patients confirmed to have previously applied ototopic medications, there were possibly others that had applied non-orthodox medications to the ears before presenting in the hospital.

The reason for disproportionate tendency of females (64.8%) to develop Otomycosis could be related to hormonal factors. Other studies had documented more predisposition of females, 13,14 but had not offered any reason nor explanation for the finding. Majority of patients were diagnosed with only one (unilateral) external auditory canal (EAC) infection. 8 The left ear was more affected than the right ear (50.7 to 35.2%). While more left ear affectation has also been documented in other similar studies,^8^,^13^ the reason for this propensity has not been elucidated. More than one-tenth of the patients (14.1%) had bilateral ear involvement, three among these (33.3%) had uncontrolled diabetes which is an immune suppressive co-morbidity. Bilateral ear involvement has been reported to be more common in the immunocompromised patients, 15 which might increase the patients' morbidity.

Drugs like Nystatin and Clotrimazole are good for treatment of Otomycosis caused by Candida species but are not effective for Aspergillus species. Hence proper identification of the causative agents is essential to minimize recurrence and complications of Otomycosis,^6^ as it guides drug choice. This study documented a positive fungal culture of 28.1%, which is within the previously reported range of 19.6% to 43.0% 9,13,16. There appeared to be a lethargy among Otologists in processing samples for fungal studies. In Denmark, less than half (42%) of ENT Consultants sent material for fungal culture and sensitivity before commencing treatment of Otomycosis. 4 This lethargy may be because the diagnosis is often clinically-obvious. The rather long processing time of up to three weeks to confirm the result, coupled with the non or irregular availability of the appropriate technology for culture, as observed at a period in the course of this study, may encourage this attitude. Abastabar et al, 17 had previously decried the lack of appropriate laboratory technology in microbiological identification of some strains of fungi in the developing countries, which is likely to underestimate the number of patients affected, and limit characterization of the strains.

Previous studies had reported that strains of Aspergillus and Candida in different proportions, were most commonly responsible for Otomycosis. 10,11,12 Pathogenic strain of fungi seen in our patients were Aspergillus and Candida species. The different strains reported had been A niger, A.fumigatus, A. flavus and C. albicans. A study that compared fungal stains based on patients' immune status, reported that Aspergillus sp were the most commonly isolated fungi in the immunocompetent group, while Candida albicans was most isolated among the immunocompromised group.^15^ Such delineation of patients according to the immune status was not performed in this study.

The clinical factors associated with positive fungal culture in this study included both the mean, and the median. ages as noted by other authors.8 A systematic review and one-way meta-analysis on treatment of Otomycosis however reported no significant relationship between the incidence of Otomycosis and age of the patients.^18^ Association of Otomycosis and age will require further clarifications with randomized controlled prospective study.

Self-ear cleaning, instillation of mustard oil and use of ear drops had been suspected to be common predisposing factors in Otomycosis.^6^ In this study however, habitual-ear cleaning was not found to be associated with a positive fungal culture. There is an almost universal practice of self-ear cleaning for hygiene, or for relieving itchy ears in our environment.^19^ Self-ear cleaning may lead to bruising of the lining of the ear canal, predisposing to Otomycosis. On the contrary, non-previous application of ototopic medications was significantly associated with a positive fungal culture. Most ototopic agents instilled are antibiotics, antifungals or steroids,^20^ which have a tendency of suppressing or masking the growth of the fungus. Consequently, some patients who genuinely had Otomycosis but had previously applied some antifungal ototopic drugs could have negative fungal culture result. Conversely, prolonged use of antibiotic ototopical drops clears normal flora in the ear canal and may encourage fungal proliferation, while prolonged steroid use in the ear may weaken skin defenses hence facilitating fungal invasion. Most routinely used ototopic drops have antibiotic/steroid/antifungal in varying combinations, however it was not practicable to obtain information on the composition of the ototopical drops used by the 22 patients in this study. The possibility that the ototopic medications used were probably of the higher concentration antifungal agents may be suggested. Non-usage of ototopic drugs allows the fungus to grow unhindered, exhibiting a full expression and can be easily cultured.

Another factor associated with positive fungal result was presence of co-morbidity in the patients. While the common co-morbidities seen had been identified, patients with uncontrolled diabetes are particularly susceptible to immunosuppression. Uncontrolled or poorly controlled diabetes affects the B and T cell immunity which increases chances of ear infections including fungal infections. The reduced immunity it causes also increases chances of fungal necrotizing otitis externa.^3^ Any patient with Otomycosis who is also diabetic, should in addition to treatment with antifungal therapy, have the blood sugar levels controlled with medical therapy to reduce the chances of complications. Theoretically, it was expected that multiple (against single) co-morbidities could be associated with positive fungal culture result. Since multiple co-morbidities may aggravate systemic depressed immunity, an expectation of multiple co-morbidities being associated with positive fungal cultures than single co-morbidity, could be justified. This was however not observed in this study.

The treatment modalities used was mainly local debridement of the ear, and topical antifungal agents' application thereafter. The local debridement methods deployed included ear syringing and ear toileting as appropriate. The debridement removed the bulk of the fungal hyphae, mycelium, crusts and other materials secreted into the external auditory canal, and created a patent canal. Patency of the canal is best checked with otomicroscopy, but simple otoscopy may suffice. Topical antifungal drugs are preferred for treatment, 4 because with a patent canal, the topical drugs would have access to the residual fungus. The common antifungal drugs used in this study were Clotrimazole or Locacorten vioformis cream. Clotrimazole has been recommended for treatment of Otomycosis because of its effectiveness in treating the infection, 21 and also reducing the relapse. 22 Locacorten has also been noted to be safe and effective, although there is insufficient evidence to support increased efficacy of either of the drugs for treatment of Otomycosis.^23^ Other antifungal agents used in other studies included topical betadine (Povidone-iodine), 10 and topical tolnaftate. 12

In this study, we discovered a generally favourable response to treatment with 91.5% of patients recording resolution of the symptoms. It had been confirmed that treatment regimens such as topical antifungals coupled with mechanical debridement are generally effective.14 However despite the generally good response to treatment, some complications like non-resolution and recurrences may occur. In this study, there were two (2.8%) non-resolutions and four (5.6%) recurrences, giving a total complication rate of 8.4%. A previous report observed relapse of disease in only 3.1% with A. niger being the main fungus. 22 Non resolutions may occur in patients with multiple pathologies as noted in one patient who had allergy, was hypertensive and had developed congestive cardiac failure. The other patient whose Otomycosis did not resolve despite appropriate treatment, had uncontrolled diabetes. Relapse and recurrence of Otomycosis may be related to inadequate treatment, with residual infection, or a re-infection when the predisposing factors re-present.

Some limitations noted in this study include its retrospective design with existence of missing data. Furthermore, because patient records were used, it was difficult to ascertain the laboratory procedures performed on individual specimens of patients. Unfortunately, specific laboratory procedures were not recorded in patient charts, thus limiting the quality of the results. The non-availability of reagents at some periods during the study is also accepted as a limitation. These limitations will however not negate the validity of the study.

In Conclusion, we found that Otomycosis commonly presented with itchy ears, ear blockage, hearing loss and otalgia, with the pathogenic fungi being Aspergillus and Candida species. The factors associated with Otomycosis were age, non-usage of ototopic agents and presence of co-morbidities. Treatment modality was local debridement and topical antifungal agents, with appreciable favourable response in majority of the patients.
